# Retrieval‐augmented generation versus document‐grounded generation: a key distinction in large language models

**DOI:** 10.1002/2056-4538.70014

**Published:** 2025-01-16

**Authors:** Shunsuke Koga, Daisuke Ono, Amrom Obstfeld

**Affiliations:** ^1^ Department of Pathology and Laboratory Medicine Hospital of the University of Pennsylvania Philadelphia PA USA

We read with great interest the article by Hewitt *et al*., ‘Large language models as a diagnostic support tool in neuropathology’ [1]. The authors effectively applied large language models (LLMs) to interpreting the WHO classification of central nervous system tumors; however, we wish to address a technical aspect of their study that warrants clarification.

The authors described their approach as retrieval‐augmented generation (RAG). Based on the methods described, the study involved attaching a Word document containing the WHO diagnostic criteria to the prompt to guide its responses. We believe that this approach is more accurately described as document‐grounded generation rather than true RAG. Document‐grounded generation refers to methods where the model generates outputs explicitly based on a preprovided document, which serves as a static reference [2]. Unlike RAG, which retrieves information dynamically from external sources [3], document‐grounded generation relies entirely on data embedded in the input prompt at the time of execution. In this study, the WHO criteria were provided with the prompt, which allowed the model to use this information without real‐time retrieval. This method is a type of in‐context learning, relying on curated contextual data embedded in the input [4].

Our own work provides an example of in‐context learning in a different domain, namely image classification. We evaluated GPT‐4 Vision (GPT‐4V) for classifying histopathological images stained with tau immunohistochemistry, including neuritic plaques, astrocytic plaques, and tufted astrocytes [5]. Although GPT‐4V initially struggled, few‐shot learning with annotated examples, which is a specific application of in‐context learning, significantly improved its accuracy, matching that of a convolutional neural network model trained on a larger dataset. These findings demonstrate the utility of in‐context learning for both text‐based and image‐based tasks, with the latter presenting unique challenges for LLMs [6].

Although in‐context learning is an effective approach, it has limitations worth considering. Since this method uses static data that are preloaded data into the prompt, errors can occur if the information is outdated or inaccurate. In‐context learning may also lead to overfitting to the given context, limiting the model's ability to generalize to other scenarios. If the contextual data are overly complex, the model might misinterpret the information or fail to generate accurate outputs [7]. To ensure reliability, it is important to carefully select the input data, update it regularly, and consider these limitations when designing tasks.

In summary, clarifying the differences between RAG, document‐grounded generation, and in‐context learning is essential, especially for readers less familiar with these concepts. Nonetheless, we support the authors' conclusion that incorporating external data improves diagnostic performance. Their study, interpreted as an example of document‐grounded generation, demonstrates how LLMs can effectively assist in medical tasks when supported by well‐curated contextual data.

## Author contributions

SK: conceptualization, drafting the manuscript. DO: reviewing and editing the manuscript. AO: reviewing and editing the manuscript.

## Conflict of interest statement

No conflicts of interest were declared.
